# Effects of cecropin antimicrobial peptides on growth and intestinal health in growing male minks

**DOI:** 10.3389/fvets.2025.1565580

**Published:** 2025-05-14

**Authors:** Jian Chen, Xiaojun Yu, Guang Wang, Ziyi Jiang, Lingpeng Kong, Huanle Zhang, Lihua Wang

**Affiliations:** College of Animal Science and Technology, Qingdao Agricultural University, Qingdao, China

**Keywords:** CAD, growing male minks, growth performance, digestibility, immunity, intestinal microflora

## Abstract

This study investigated the effects of dietary supplementation with cecropin antimicrobial peptides (CAD) on growth performance and intestinal health in growing male minks (*Neovison vison*). A cohort of 60 male minks (65 days old) were evenly divided into six groups and fed a basal diet supplemented with CAD at 0 (control), 100, 200, 300, 400, or 500 mg/kg for 8 weeks. The findings revealed that the minks in 200 mg/kg CAD group had greater growth performance, with significantly higher final body weight (FBW) and average daily gain (ADG). Compared to the minks in the control (*p* < 0.05). Digestibility analyses at week 3 demonstrated that CAD supplementation enhanced ether extract (EE) digestibility (*p* < 0.05), while 200, 400, and 500 mg/kg CAD improved crude protein (CP) digestibility (*p* < 0.05). Intestinal morphology assessments indicated that 200 mg/kg CAD significantly increased duodenal and jejunal villus height (both *p* < 0.05) and jejunal villus height-to-crypt depth ratio (*p* < 0.05) compared to the control. Serum immunological analyses revealed elevated levels of complement C4 and IgG in CAD-supplemented groups (*p* < 0.05). Notably, the 100 mg/kg CAD group exhibited the higher serum IgA, IgM, and complement C3, and less jejunal TNF-*α* levels (all *p* < 0.05). Microbiota profiling showed that CAD supplementation reduced the relative abundance of *Escherichia-Shigella* and *Mycoplasma*, while 100, 200 and 400 mg/kg CAD decreased *Peptostreptococcaceae* populations (*p* < 0.05). The 100 mg/kg CAD group displayed optimal immune enhancement and microbiota modulation, whereas the 200 mg/kg group achieved the best growth performance and intestinal function. These results suggest that dietary CAD supplementation at 100–200 mg/kg effectively improves growth, nutrient utilization, and intestinal health in growing male minks.

## 1 Introduction

Over the past few decades, antibiotics have been pivotal in livestock farming, serving as growth promoters that enhance animal growth, improve feed efficiency, and reduce the incidence of bacterial infections (1, 2). However, the excessive and improper use of these antimicrobial agents has contributed to the antibiotic-resistant pathogens, presenting a significant threat to both animal health and public safety (3, 4). In response to this issue, the EU, the United States, China, and several other regions have banned the use of antibiotics as growth promoters in animal feed. Consequently, there is an urgent and extensive need for research focused on identifying and developing effective alternatives to antibiotics (5).

Cecropin antimicrobial peptides (CAD), a subclass of antimicrobial peptides (AMPs), are increasingly recognized as a potential substitute for conventional antibiotics. AMPs are crucial components of the innate immune defense systems in various organisms and typically consist of amino acid sequences ranging from 7 to 100 residues (6). These small molecular weight peptides exhibit a range of bioactivities, including antibiofilm, antifungal, antiviral, and immunomodulatory effects (7). They interact directly with the bacterial cell membrane, initiating metabolite leakage, cell lysis, or disrupting the electrochemical ion gradient, which ultimately leads to cell death (8). Some AMPs can also penetrate bacterial or nuclear membranes to interfere with essential cellular functions, including enzymatic activities, cell wall synthesis, nucleic acid replication, and protein synthesis, thereby hastening the elimination of microorganisms (9). The unique mechanisms of action of AMPs are associated with lower toxicity and a reduced likelihood of resistance development, which confers on them significant advantages over conventional antibiotics (10, 11). Furthermore, AMPs possess immunomodulatory capabilities that facilitate the elimination of pathogens through the modulation of cellular immune responses (12).

The first well-documented AMPs were cecropins, discovered and characterized in the giant silk moth *Hyalophora cecropia* (13). Research indicates that CADs can reduce populations of harmful bacteria such as *Escherichia coli* in weaned piglets while simultaneously increasing the presence of beneficial *Lactobacilli* (14). CADs also impact intestinal morphology by altering intestinal villi height and crypt depth, which promotes the growth of intestinal villi essential for nutrient absorption (15). These peptides modulate the body’s levels of inflammatory mediators, thereby reducing the inflammatory response in the intestinal tissue (16). Previous studies have demonstrated that CADs can enhance immune function and promote intestinal health by improving intestinal morphology and the balance of gut microbiota. Consequently, nutrient digestibility and animal performance are improved (14).

While CADs have been demonstrated to enhance immune responses and improve intestinal homeostasis in several species (15, 17–19), their application in minks remains largely unexplored. To evaluate the effects of CADs on growth performance, nutrient digestibility, serum immunity, and intestinal health in minks, in this study we compared these parameters in minks that were supplemented with CADs with those that were not supplemented during the feeding trial period.

## 2 Materials and methods

### 2.1 Animals, experimental design, and diets

The study was carried out at a commercial mink ranch. Sixty healthy black male minks at 65 days of age were selected for the study. These minks were evenly assigned to one of six groups (n = 10). Based on previous studies in poultry and fish (16, 20–23), the minks were fed a basal diet supplemented with CAD at 0 (control), 100, 200, 300, 400, and 500 mg/kg of diet, respectively. The CAD (Cecropin Antimicrobial Peptides, with activity ≥ 1 million IU/g and purity ≥ 98%) used in the experiment was sourced from Zhongnong Yingtai Biotechnology Co., Ltd., Beijing, China.

Throughout the 8-week study period, these minks were housed in individual metal wire cages under natural light conditions. During the experimental period, the minks had *ad libitum* access to water via a drinker in their home cages. The sticky fresh diets were delivered to the top of the cages twice daily (at 5:00 AM and 5:00 PM), providing the minks with approximately *ad libitum* access to the diets. The basal diet was formulated at the farm in accordance with standard commercial guidelines. The specific ingredients and nutritional content of the diets are detailed in Table 1.

**Table 1:** Composition and nutrient levels of basal diets (DM basis, %).

Items	1–4 weeks	5–8 weeks
Ingredients		
Duck skeleton	18.00	18.00
Chicken intestines	15.00	15.00
Steamed egg	15.00	10.00
Rough fish	10.00	10.00
Cod bone	10.00	10.00
Anglerfish head	10.00	10.00
Extruded corn	9.50	9.50
Chicken skeleton	5.00	5.00
Extruded soybean	2.00	2.00
Pig blood meal	1.50	1.50
Fish meal	1.00	1.00
Soybean oil	0.00	5.00
Premix^2^	3.00	3.00
Total	100.00	100.00
Nutrient levels		
ME (MJ/kg)^3^	16.48	17.81
EE	18.09	24.41
CP	35.04	30.90
Ash	11.80	11.75
Ca	2.55	2.83
TP	1.18	1.01

1 The nutritional level is based on the dry matter. The measured dry matter content of the diet for 1 to 5 weeks is 35.33%, and for 6 to 8 weeks it is 38.00%.

2 The premix provided the following per kg of the diets (DM basis): Met 5,000 mg, Lys 5,000 mg, NaCl 5,000 mg, CaHPO4 5,000 mg, VA 10000 IU, VC 120 mg, VE 150 mg, VK3 1 mg, VB1 20 mg, VB2 10 mg, VB6 10 mg, VB12 0.1 mg, nicotinic acid 40 mg, pantothenic acid 13 mg, folic acid 1 mg, biotin 0.5 mg, Fe 60 mg, Zn 50 mg, Mn 30 mg, Cu 5 mg, I 0.5 mg, Se 0.4 mg.

3 ME was calculated using the formula by compound feed of mink (LS/T 3403–1992) and the equation: ME = (0.85 × CP% × 4.5 + 0.90 × %EE × 9.5 + 0.75 × NFE% × 4.0) × 4.184, NFE(%) = 100-CP(%)-EE(%)-Ash(%).

### 2.2 Measurements

#### 2.2.1 Growth performance

All minks in the six groups were individually weighed at week 0 and week 8 to determine initial body weight (IBW), FBW, and ADG. The daily feed intake of minks was monitored. The diet provided to each mink was weighed and recorded. Any leftover diet was collected and weighed before the next feeding session. Based on these data, the average daily feed intake (ADFI) and the feed-to-gain ratio (F/G) were calculated. Six minks from each group at week 8 of the study were selected to measure body size, which was determined by the length from the base of the tail to the tip of the nose.

#### 2.2.2 Apparent nutrient digestibility

Nutrient digestibility was determined using the endogenous indicator method during a digestion experiment. Six replicates from each group were selected for analysis. Fresh feces and feed were sampled from all groups at week 3 and 7 of the study and pooled over a three-day period. The samples were then dried, ground, and sieved for analysis. The concentrations of hydrochloric acid insoluble ash, dry matter (DM), crude protein (CP), ether extracts (EE), calcium (Ca), and phosphorus (P) were determined in both fecal and feed samples. Apparent digestibility of these nutrients was then calculated based on the measured concentrations.

#### 2.2.3 Biochemical and immunological indicators in serum

Six minks per group were selected at the conclusion of the study for blood sample collection via cardiac puncture. These blood samples were centrifuged at 3500 × g for 10 min at 4°C to separate the serum, which was analyzed for the levels of total protein (TP), aspartate aminotransferase (AST), albumin (ALB), alanine aminotransferase (ALT), blood urea nitrogen (BUN), lysosomal enzyme (LZM), immunoglobulin A (IgA), immunoglobulin M (IgM), immunoglobulin G (IgG), C3, and C4. All analyses were performed using a double antibody sandwich enzyme-linked immunosorbent assay (ELISA) kit manufactured by Enzyme-Linked Biotechnology Co. Ltd. (Shanghai, China).

#### 2.2.4 Intestinal immunological indicators and morphology

At the end of the experiment, intestinal samples were collected from six euthanized minks per group, including 2–3 cm segments from the mid-duodenum and mid-jejunum. The samples were rinsed with phosphate-buffered saline (PBS), blotted dry, and stored at −80°C for further analyses. Additionally, approximately 1 g of jejunum tissue was excised for further analysis. The segments of the duodenum and jejunum were fixed in a 10% formaldehyde solution. After fixation, the intestinal segments were prepared into intestinal tissue slides through a series of histological processing steps, including trimming, washing, dehydration, clarification, wax impregnation, embedding, sectioning, and staining. The ZEN 2011 (Blue edition) software was employed to measure the VH and CD. Based on these measurements, the V/C ratio was then calculated.

The jejunum samples were homogenized with 9 mL of PBS at pH 7.4, followed by centrifugation at 3500 × g for 10 min to separate the supernatant. The supernatant from the homogenized jejunum tissue was utilized with a double antibody sandwich enzyme-linked immunosorbent assay (ELISA) kit produced by Enzyme-Linked Biotechnology Co., Ltd. (Shanghai, China), to measure the levels SIgA, TNF-*α*, IFN-*γ*, IL-1β, IL-8, IL-10, and lysozyme (LZM). A full-wavelength enzyme labeling instrument (CFX96, Bohle, United States) was utilized to determine the concentrations of these components.

#### 2.2.5 Intestinal microbiota

Rectal intestinal contents were collected from six minks per group and stored at −80°C. Genomic DNA was extracted from these samples using the Fast DNA Spin Kit for Soil (MP Biomedicals, United States), following the method described by Jin et al. (24). The integrity of the extracted DNA was verified via 1% agarose gel electrophoresis. The V3-V4 hypervariable region of the bacterial 16S rRNA gene was amplified by PCR using the universal primers 338F and 806R. PCR reactions were performed on an ABI GeneAmp® 9,700 thermocycler. The program included an initial denaturation at 95°C for 3 min, followed by 27 cycles of denaturation at 95°C for 30 s, annealing at 55°C for 30 s, and extension at 72°C for 30 s. A final extension step was included at 72°C for 10 min. The PCR products were visualized on a 2% agarose gel, purified using the AxyPrep DNA Gel Recovery Kit (Axygen, United States), and confirmed by electrophoresis on a 2% agarose gel. The purified DNA was quantified using the QuantiFluor™-ST Blue Fluorescence Quantification System (Promega, United States). An Illumina MiSeq sequencing library was prepared from the purified amplicons according to the manufacturer’s protocols and sequenced on the Illumina MiSeq platform using the PE 300 protocol.

### 2.3 Statistical analysis

All statistical analyses were performed using SPSS 25. 0 (SPSS Institute Inc., Chicago, United States). All data sets were tested for normal distribution using the Univariate Procedure of the SPSS. Since the data for ADFI and F/G were not normally distributed, non-parametric analysis was conducted using the Kruskal-Wallis test. Normally distributed data were evaluated using the ANOVA procedure in SPSS 25.0 to examine the effects of CAD. Duncan’s test was used to separate means.

Additionally, the correlation analysis between immunity, apparent nutrient digestibility, and the intestinal microbiota of minks was performed using the Spearman method on the I-Sanger cloud platform.

## 3 Results

### 3.1 Growth performance

CAD significantly affected the FBW and ADG of growing males (both *p* < 0.05; Table 2). Compared to the minks in the control, those in the CAD group at 200 mg/kg of diet exhibited a significant increase in FBW and ADG (both *p* < 0.05).

**Table 2:** Effects of CAD on growth performance of growing male minks.

Items	Supplemental levels/(mg/kg)						*p*-value
	0	100	200	300	400	500	
Initial weight/g	681.75 ± 82.73	722.63 ± 72.02	713.50 ± 80.48	701.00 ± 101.82	696.75 ± 78.25	701.13 ± 103.22	0.95
Final weight/g	1847.75 ± 221.84^bc^	2029.38 ± 127.06^ab^	2099.13 ± 172.56^a^	2035.20 ± 211.83^ab^	2029.75 ± 154.32^ab^	1803.25 ± 241.84^c^	0.02
ADG/g	17.67 ± 3.07^bc^	19.80 ± 2.67^abc^	20.99 ± 3.25^a^	20.22 ± 2.66^ab^	20.20 ± 2.22^ab^	16.70 ± 2.86^c^	0.03
ADFI/g	241.41 ± 14.63	242.55 ± 10.01	239.34 ± 22.09	245.53 ± 14.45	254.75 ± 5.57	241.73 ± 10.71	0.29
F/G	14.10 ± 2.98	12.48 ± 2.00	11.69 ± 2.47	12.25 ± 1.05	12.75 ± 1.47	14.78 ± 2.11	0.06
Body length/cm	42.83 ± 1.94	44.00 ± 1.55	43.50 ± 2.26	43.50 ± 1.05	44.25 ± 0.69	43.58 ± 2.29	0.79

In the same row, values with no letter or the same letter superscripts mean no significant difference (*p* > 0.05), while with different small letter superscripts mean significant difference (*p* < 0.05).

### 3.2 Nutrient apparent digestibility

CAD affected the apparent digestibility of CP and EE in male minks only at week 3 of the study (both *p* < 0.05; Table 3). At week 3 of the study, the CAD groups exhibited greater digestibility of EE (*p* < 0.05), and the CAD groups at 200, 400, and 500 mg/kg had greater digestibility of CP (*p* < 0.05) than the control.

**Table 3:** Effects of CAD on nutrient apparent digestibilities of growing male minks %.

Items	Supplemental levels/(mg/kg)						*p*-value
	0	100	200	300	400	500	
Week 3							
DM	76.11 ± 0.53	75.31 ± 0.64	75.86 ± 0.30	75.42 ± 0.85	76.63 ± 1.11	75.83 ± 1.22	0.16
CP	79.41 ± 1.67^c^	79.43 ± 1.41^c^	82.45 ± 0.90^a^	80.56 ± 1.16^bc^	82.40 ± 1.76^a^	82.08 ± 1.06^ab^	<0.01
EE	84.66 ± 2.25^b^	87.77 ± 2.50^a^	90.13 ± 2.10^a^	88.08 ± 1.19^a^	89.11 ± 1.77^a^	89.33 ± 1.84^a^	<0.01
Ca	28.38 ± 3.63	27.91 ± 4.48	28.26 ± 2.95	29.31 ± 2.16	28.65 ± 4.12	29.74 ± 4.00	0.96
P	25.51 ± 4.37	27.69 ± 2.5	27.70 ± 3.34	27.89 ± 4.01	25.72 ± 4.26	24.94 ± 5.37	0.71
Week 7							
DM	72.79 ± 1.44	71.36 ± 1.32	72.95 ± 2.12	73.54 ± 1.73	73.31 ± 1.90	71.40 ± 1.07	0.13
CP	75.16 ± 2.77	75.11 ± 1.47	75.68 ± 2.07	75.29 ± 3.87	75.35 ± 2.18	74.09 ± 4.37	0.97
EE	91.39 ± 3.16	92.98 ± 1.55	89.33 ± 2.44	91.59 ± 2.52	91.84 ± 0.97	90.69 ± 1.97	0.18
Ca	25.56 ± 1.80	23.57 ± 5.46	23.83 ± 3.92	24.83 ± 5.65	24.00 ± 3.52	25.02 ± 4.53	0.97
P	42.60 ± 3.42	38.86 ± 2.80	38.64 ± 2.68	40.84 ± 3.63	41.27 ± 2.53	42.25 ± 3.11	0.19

In the same row, values with no letter or the same letter superscripts mean no significant difference (*p* > 0.05), while with different small letter superscripts mean significant difference (*p* < 0.05).

### 3.3 Intestinal morphology

CAD significantly influenced duodenal and jejunal VH, and jejunal V/C ratio in growing male minks (all *p* < 0.05; Table 4). The CAD group at 200 mg/kg increased both duodenal and jejunal VH, as well as the jejunal V/C (all *p* < 0.05) than the control. Additionally, the CAD group supplemented with 100 mg/kg had a greater jejunal VH (*p* < 0.05), and the CAD group supplemented with 500 mg/kg had a greater jejunal CD (*p* < 0.05) compared with the control.

**Table 4:** Effects of CAD on intestinal morphology of growing male minks.

Items	Supplemental levels/(mg/kg)						*p*-value
	0	100	200	300	400	500	
Duodenum							
Villus height/μm	941.85 ± 48.48^b^	967.06 ± 48.34^b^	1196.64 ± 138.9^a^	884.58 ± 41.54^b^	1014.41 ± 36.84^b^	967.07 ± 15.80^b^	<0.01
Crypt depth/μm	570.05 ± 19.90	627.55 ± 123.14	516.06 ± 167.30	615.04 ± 20.90	641.24 ± 155.54	463.45 ± 70.07	0.42
V/C	1.66 ± 0.13	1.59 ± 0.30	2.53 ± 0.89	1.44 ± 0.05	1.68 ± 0.53	2.11 ± 0.35	0.05
Jejunum							
Villus height/μm	902.70 ± 110.91^b^	1165.91 ± 138.17^a^	1137.17 ± 17.28^a^	945.90 ± 46.06^b^	1036.70 ± 79.77^ab^	1032.43 ± 121.49^ab^	<0.01
Crypt depth/μm	503.94 ± 61.72^bc^	563.88 ± 66.40^ab^	478.04 ± 59.19^bc^	429.92 ± 90.92^c^	466.36 ± 43.06^bc^	633.48 ± 142.42^a^	<0.01
V/C	1.82 ± 0.32^bc^	2.09 ± 0.35^abc^	2.41 ± 0.33^a^	2.26 ± 0.39^ab^	2.23 ± 0.15^abc^	1.75 ± 0.61^c^	<0.01

In the same row, values with no letter or the same letter superscripts mean no significant difference (*p* > 0.05), while with different small letter superscripts mean significant difference (*p* < 0.05).

### 3.4 Measurement of serum samples

#### 3.4.1 Serum biochemical parameters

CAD significantly influenced the serum ALB levels in growing male minks (*p* < 0.05; Table 5). Compared to the control, CAD at 100, 300, 400, and 500 mg/kg increased serum ALB levels (*p* < 0.05).

**Table 5:** Effects of CAD on serum biochemical indices of growing minks.

Items	Supplemental levels/(mg/kg)						*p*-value
	0	100	200	300	400	500	
ALB(G/L)	33.82 ± 2.37^c^	57.27 ± 3.27^a^	35.68 ± 3.08^c^	47.07 ± 4.01^b^	46.58 ± 5.50^b^	46.83 ± 2.12^b^	<0.01
ALT(U/L)	67.88 ± 1.58	67.92 ± 2.32	64.94 ± 3.25	65.51 ± 1.78	68.36 ± 1.72	66.89 ± 4.96	0.24
AST(U/L)	55.13 ± 3.65	57.06 ± 2.43	55.02 ± 2.92	57.04 ± 2.90	58.68 ± 2.29	57.47 ± 4.51	0.35
BUN(mmol/L)	15.52 ± 0.77	16.45 ± 0.97	16.46 ± 0.68	16.45 ± 0.80	16.66 ± 2.20	15.23 ± 0.96	0.21
TP(G/L)	96.28 ± 4.52	100.71 ± 5.66	99.44 ± 6.40	96.92 ± 6.85	102.66 ± 4.70	96.96 ± 7.65	0.41
LZM(U/mL)	625.91 ± 107.04	618.49 ± 138.30	575.34 ± 144.54	511.55 ± 105.73	478.84 ± 160.88	495.19 ± 73.03	0.20

In the same row, values with no letter or the same letter superscripts mean no significant difference (*p* > 0.05), while with different small letter superscripts mean significant difference (*p* < 0.05).

#### 3.4.2 Serum immune indices

CAD significantly influenced the serum levels of C3, C4, IgA, IgG, and IgM in growing male minks (all *p* < 0.05; Table 6). Compared to the control, CAD increased serum C4 and IgG levels (both *p* < 0.05), in particular, CAD at 100 mg/kg increased serum IgA, IgM, and C3 levels (all *p* < 0.05).

**Table 6:** Effects of CAD on immune indices of growing minks.

Items	Supplemental levels/(mg/kg)						*p*-value
	0	100	200	300	400	500	
C3/(μg/mL)	81.50 ± 9.89^d^	160.87 ± 13.59^a^	94.97 ± 9.63^d^	137.81 ± 10.37^b^	116.64 ± 64.00^c^	127.22 ± 13.24^bc^	<0.01
C4/(μg/mL)	195.04 ± 23.43^d^	337.32 ± 24.52^a^	218.79 ± 21.08^c^	289.25 ± 7.19^b^	281.73 ± 15.86^b^	285.49 ± 7.98^b^	<0.01
A IgA/(g/L)	0.18 ± 0.02^d^	0.34 ± 0.02^a^	0.21 ± 0.01^d^	0.29 ± 0.03^b^	0.25 ± 0.02^c^	0.27 ± 0.03^bc^	<0.01
G IgG/(g/L)	13.18 ± 1.92^e^	30.24 ± 2.59^a^	17.64 ± 2.13^d^	24.50 ± 1.28^b^	20.90 ± 1.73^cd^	22.70 ± 2.20^bc^	<0.01
M IgM/(g/L)	2.71 ± 0.18^c^	4.65 ± 0.25^a^	2.95 ± 0.30^c^	4.00 ± 0.32^b^	3.72 ± 0.29^b^	3.86 ± 0.27^b^	<0.01

In the same row, values with no letter or the same letter superscripts mean no significant difference (*p* > 0.05), while with different small letter superscripts mean significant difference (*p* < 0.05).

### 3.5 Jejunum immune indices

CAD significantly affected the levels of SIgA, TNF-*α*, and IL-10 in the jejunum of growing male minks (all *p* < 0.05; Table 7). Compared to the control, CAD at 100, 200, 500 mg/kg increased the jejunum SIgA level (*p* < 0.05), CAD at 200 and 400 mg/kg increased jejunum IL-10 level (*p* < 0.05), and CAD at 100 mg/kg reduced TNF-α levels (*p* < 0.05).

**Table 7:** Effects of antimicrobial peptide on jejunal immune indexes of growing male minks.

Items	Supplemental levels/(mg/kg)						*p*-value
	0	100	200	300	400	500	
SIgA/(μg/mL)	34.48 ± 5.03^cd^	43.20 ± 4.07^a^	40.95 ± 4.62^ab^	35.00 ± 4.43^bcd^	31.12 ± 2.97^d^	38.43 ± 7.47^abc^	0.01
TNF-α/(pg/mL)	75.84 ± 5.37^a^	67.04 ± 2.64^b^	76.40 ± 5.15^a^	71.05 ± 4.58^ab^	76.92 ± 2.98^a^	71.75 ± 5.41^ab^	0.01
IFN-r/(pg/mL)	665.40 ± 145.44	591.75 ± 141.39	632.42 ± 118.16	650.40 ± 142.54	579.62 ± 161.68	548.38 ± 124.54	0.72
IL-1β/(pg/mL)	105.69 ± 6.84	106.77 ± 12.85	108.96 ± 9.67	113.35 ± 9.170	112.02 ± 6.73	114.64 ± 6.20	0.43
IL-8/(pg/mL)	162.51 ± 14.60	151.59 ± 5.09	163.46 ± 15.81	160.14 ± 17.53	161.01 ± 13.18	166.3 ± 7.45	0.65
IL-10/(pg/mL)	77.27 ± 3.83^bc^	78.54 ± 2.99^bc^	84.07 ± 1.25^a^	80.40 ± 2.92^ab^	82.90 ± 3.78^a^	75.44 ± 3.25^c^	<0.01
LZM/(ng/mL)	32.45 ± 1.15	30.65 ± 1.31	32.28 ± 0.57	31.09 ± 2.32	32.04 ± 3.33	29.95 ± 2.14	0.22

In the same row, values with no letter or the same letter superscripts mean no significant difference (*p* > 0.05), while with different small letter superscripts mean significant difference (*p* < 0.05).

### 3.6 Intestinal microbiota

#### 3.6.1 Alpha diversity

CAD had significant effects on the Ace and Chao1 indices in growing male minks (both *p* < 0.05; Table 8). Minks in the 100 mg/kg CAD group had greater Ace and Chao1 indices than those in the control (*p* < 0.05).

**Table 8:** Effects of antimicrobial peptide on the alpha diversity index of growing male minks.

Items	Supplemental levels/(mg/kg)						*p*-value
	0	100	200	300	400	500	
Shannon index	2.23 ± 0.51	2.60 ± 0.64	2.00 ± 0.52	1.92 ± 0.41	2.51 ± 0.67	1.98 ± 0.55	0.28
Simpson index	0.23 ± 0.10	0.22 ± 0.13	0.37 ± 0.19	0.38 ± 0.11	0.21 ± 0.13	0.32 ± 0.15	0.23
Ace index	301.87 ± 89.75^b^	464.7 ± 126.80^a^	316.61 ± 20.29^b^	246.93 ± 21.32^b^	308.90 ± 116.39^b^	388.69 ± 129.31^ab^	0.04
Chao1 index	305.25 ± 101.07^b^	462.77 ± 120.31^a^	318.18 ± 27.29^b^	236.12 ± 37.81^b^	324.96 ± 124.72^b^	365.10 ± 107.68^ab^	0.04

In the same row, values with no letter or the same letter superscripts mean no significant difference (*p* > 0.05), while with different small letter superscripts mean significant difference (*p* < 0.05).

#### 3.6.2 Intestinal microbiota

At the genus level, the relative abundance of intestinal microbiota in the six groups that exceeded 1% included *Burkholderia*, *Peptostreptococcaceae*, *Lactobacillus*, *Clostridium*, and other genera. CAD significantly altered the relative abundance of intestinal *Escherichia-Shigella*, *Mycoplasma*, *Staphylococcus*, and *Peptostreptococcaceae* in male mink rectum at the genus level (all *p* < 0.05; Table 9). Compared to the control, the CAD groups showed lower relative abundance of *Escherichia-Shigella* and *Mycoplasma* (*p* < 0.05), the CAD groups at 100, 200, and 400 mg/kg exhibited a reduced relative abundance of intestinal *Peptostreptococcaceae* (*p* < 0.05), and the CAD group at 400 mg/kg displayed a higher relative abundance of *Staphylococcus* (*p* < 0.05).

**Table 9:** Effects of CAD on relative abundance of rectal flora at genus levels of growing male minks %.

Items	Supplemental levels/(mg/kg)						*p*-value
	0	100	200	300	400	500	
*Actinomyces*	2.56 ± 5.04	13.06 ± 12.42	2.99 ± 3.13	2.59 ± 3.81	5.23 ± 5.55	9.72 ± 12.26	0.16
*Burkholderia*	18.02 ± 17.50	18.86 ± 18.98	27.46 ± 26.18	30.79 ± 33.42	32.34 ± 20.77	18.12 ± 28.6	0.83
*Escherichia-Shigella*	5.66 ± 7.70^a^	0.09 ± 0.14^b^	0.78 ± 1.59^b^	0.23 ± 0.37^b^	0.39 ± 0.83^b^	0.02 ± 0.04^b^	0.04
*Mycoplasma*	17.89 ± 22.14^a^	0.51 ± 0.83^b^	2.70 ± 6.58^b^	0.02 ± 0.02^b^	0.10 ± 0.11^b^	0.52 ± 0.65^b^	0.02
*Lactobacillus*	10.78 ± 18.11	8.91 ± 15.30	8.26 ± 17.88	12.13 ± 20.68	11.56 ± 17.25	15.75 ± 28.79	0.99
*Clostridium*	5.47 ± 7.17	13.35 ± 28.85	13.79 ± 31.18	0.53 ± 0.48	0.20 ± 0.33	16.91 ± 22.81	0.55
*Staphylococcus*	2.35 ± 2.96^b^	3.86 ± 4.05^b^	2.49 ± 2.76^b^	5.25 ± 7.88^b^	17.86 ± 12.45^a^	1.72 ± 1.34^b^	0.01
*Peptostreptococcaceae*	26.70 ± 18.2^a^	1.58 ± 3.31^b^	5.17 ± 10.24^b^	25.82 ± 23.55^a^	0.81 ± 1.72^b^	32.39 ± 19.94^a^	<0.01

In the same row, values with no letter or the same letter superscripts mean no significant difference (*p* > 0.05), while with different small letter superscripts mean significant difference (*p* < 0.05).

#### 3.6.3 Correlation analysis

**Figure 1:**
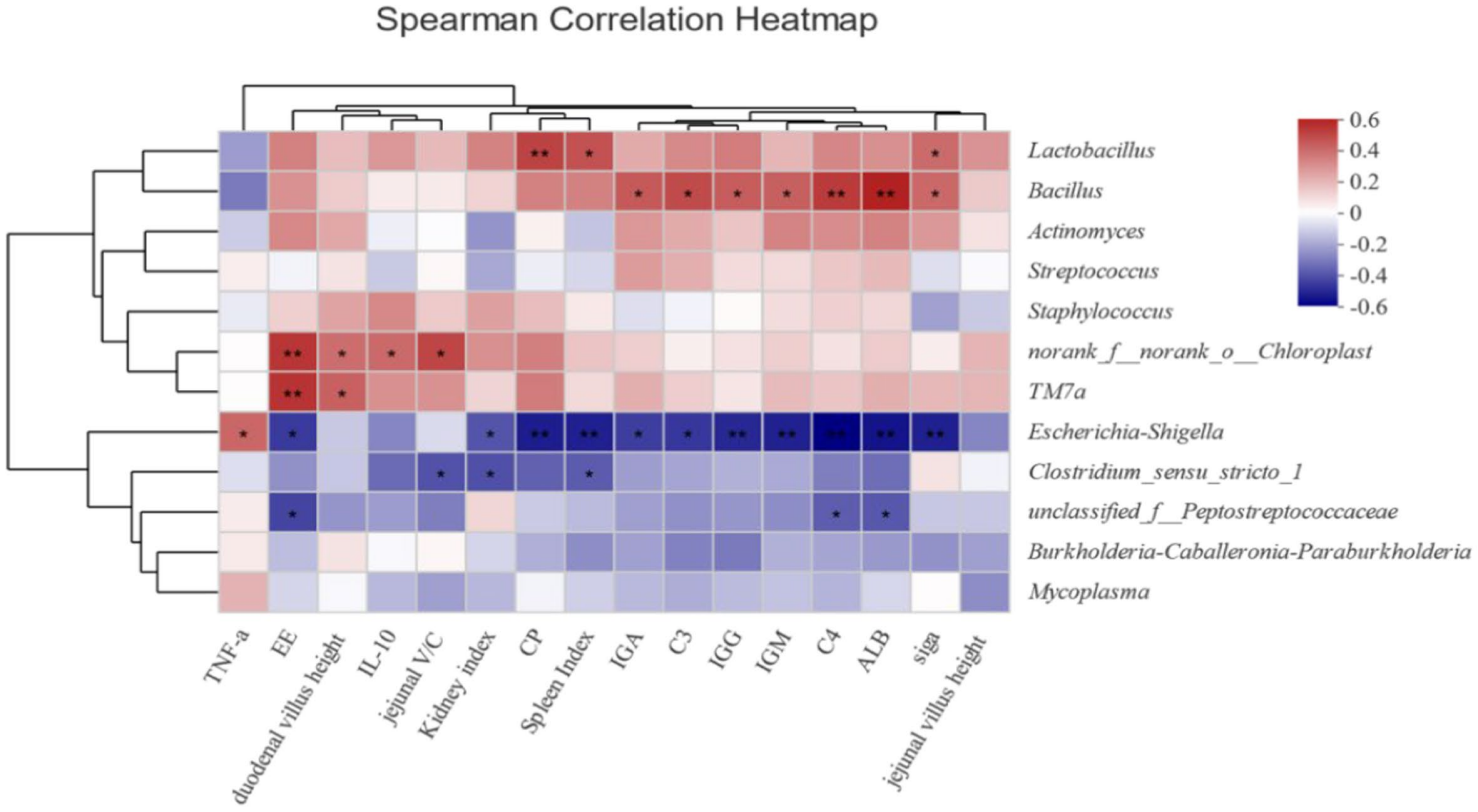
Heatmap of correlations between intestinal flora and nutrient digestibility of growing male minks (at genus level). Heatmap shows the correlation between intestinal flora (genus level) and intestinal immune function, intestinal morphology, apparent nutrient digestibility, and serum immune indicators. The X-axis and Y-axis are environmental factors and species respectively, and the correlation R value and *p* value are obtained by calculation. R-values are shown in different colors in the graph. If the *p*-value is less than 0.05, it is marked with *; if the *p*-value is less than 0.01, it is marked with **. The legend on the right is the color interval of different R-values.

Correlation analysis was conducted to evaluate the correlations between nutrient apparent digestibility, as well as related immune indices intestinal dominant bacteria and in growing male minks (Figure 1). It was found that *Escherichia-Shigella* had a negative correlation with the digestibility of EE and CP (all *p* < 0.05). Additionally, *Peptostreptococcaceae* exhibited a significantly negative correlation with digestibility of EE (*p* < 0.05).

## 4 Discussion

The current study indicated that dietary supplementation with CAD significantly influenced the growth performance in growing male minks. In particular, the minks in the 200 mg/kg CAD group had greater FBW and ADG than those minks in the control. The increased ADG was not related to ADFI, but may be associated with increased feed utilization, as CAD tended to influence the F/G in growing male minks. These results are in concordance with previous studies by Choi et al. (25) in broilers and Shi et al. (26) in piglets. Liu et al. (27) discovered that the AMPs in the diets inhibited *Escherichia coli*, thereby enhancing intestinal function and post-weaning growth performance in pigs. The intestine is a crucial site for digestion, absorption, and immunity, making the maintenance of intestinal health essential for these processes (28). AMPs can enhance the capacity for digestion and absorption by increasing the intestinal surface area, thereby promoting productive performance (29). However, high-dose AMPs may cause immunotoxicity and microbiota dysbiosis, as prior studies have demonstrated suppressed lymphocyte function (30). Our results with 500 mg/kg CAD supplementation show comparable dose-related effects, underscoring the need for optimized delivery strategies to balance therapeutic efficacy and safety. Future research should explore methods to enhance the benefits of AMPs while minimizing potential adverse effects.

The findings from the digestion experiment conducted at week 3 of the study indicated that dietary supplementation with CAD enhanced the digestibility of EE, and dietary supplementation with CAD at 200, 400, and 500 mg/kg improved the apparent digestibility of CP. However, the digestion experiment at week 7 of the study did not reveal any improvements in the apparent digestibility of nutrients due to supplementation with CAD. These findings suggest that the effects of CAD may not be significant due to the maturation of the mink’s gastrointestinal tract with age. Yoon et al. (31) found that pigs fed diets with AMP exhibited greater apparent digestibility of CP compared to those on a control diet. Wang et al. (32) and Shim et al. (33) have suggested that AMP can alter intestinal morphology, increase the nutrient contact area, and enhance intestinal enzyme activity, thereby promoting nutrient utilization. Furthermore, Rew and Rozek (34) proposed that AMPs improved nutrient digestibility by inhibiting the growth and metabolism of harmful bacteria. The correlation analysis in this study identified a significantly negative association between *Escherichia-Shigella* and the digestibility of both EE and CP, and a similar association for *Peptostreptococcaceae* with EE digestibility. Therefore, AMPs may improve nutrient digestibility by optimizing intestinal function and enhancing enzyme activity.

Enhancement in VH within the small intestine can enhance the surface area available for nutrient absorption. The V/C is indicative of the villi’s nutrient absorptive capacity (35). In this experiment, CAD at 100 mg/kg in the diet increased jejunal VH, while CAD at 500 mg/kg in the diet enhanced jejunal CD. CAD at 200 mg/kg in the diet specifically resulted in increased duodenal and jejunal VH, as well as an improved V/C. These results are consistent with findings by Liu et al. (27) in weaning pigs and Bao et al. (22) in broilers. AMPs predominantly accumulate in the jejunum and ileum, where they stimulate the proliferation of intestinal epithelial cells. This stimulation promotes the growth of villi in the jejunum and ileum, thereby increasing the V/C (36). It is concluded that dietary supplementation with CAD can promote intestinal development and improve intestinal structure.

Serum biochemical indicators are essential for assessing animal health, as they offer insights into organ function, nutritional status, and metabolic activity (37). The concentration of serum ALB can reflect the intake and utilization of protein (38). The experimental results showed that dietary CAD at 100, 300, 400, and 500 mg/kg increased serum ALB content in growing male minks. Zhan (39) also found that dietary supplementation with AMP raised serum ALB content in weaned piglets. Elevated serum ALB levels suggest improved absorption of amino acids and proteins by the organism. Furthermore, higher ALB content contributes to better nutritional support and increased antibody production, thereby stimulating immunity (40). However, serum ALB levels were reduced at the 200 mg/kg CAD supplementation level. This decrease may stem from multiple factors. AMPs, with their broad-spectrum antibacterial activity, can disrupt the gut microbiota, potentially leading to secondary infections due to the loss of microbiota’s protective effects, as suggested by Su (41). Additionally, research indicates that AMPs may be unstable under various physiological conditions, including exposure to proteases, serum, salt, or pH fluctuations (42). These factors could potentially explain the reduced ALB levels observed at the 200 mg/kg CAD supplementation.

The current study indicated that dietary supplementation with CAD increased the serum C4 and IgG contents. Additionally, dietary supplementation with CAD at 100 mg/kg significantly elevated the serum C3, IgM, and IgA contents. Similar findings have been reported in chickens, where dietary supplementation with AMPs increased serum levels of IgA, IgG, IgM, and C3 (43, 44). Shan et al. (45) also showed that dietary supplementation with AMP significantly raised the serum levels of IgG, IgA, IgM, and C4 in piglets. The serum immunoglobulins serve as an indicator of the body’s immune function. These immunoglobulins also play a protective role against pathogenic viruses and microorganisms in the extravascular compartment (46). Immunoglobulins possess the capability to neutralize toxins and prevent pathogen infections by binding specifically to their corresponding antigens (47). The complement system serves as an enhancer or cofactor for antibody molecules and is pivotal in humoral immune regulation, defense mechanisms, and immunopathological processes (48). Consequently, C3 levels can reflect the total serum complement activity, which is a critical indicator for evaluating humoral immunity.

AMPs have the potential to modulate immune responses through various pathways, including toll-like receptor signaling, NF-κB, and MAPK signaling pathways (49). Lee E et al. (50) utilized Western blotting to investigate the expression of COX-2 and MAPK, revealing that CAD inhibits intracellular signaling via the ERK, JNK, and p38 MAPK pathways, thus serving as a preventive and therapeutic agent for inflammatory diseases. Kim et al. (51) demonstrated that AMPs suppress LPS-induced TLR4 and NF-κB expression, indicating that papiliocin influences inflammatory responses through its effects on the TLR4/NF-κB pathway. Furthermore, CAD exhibits anti-inflammatory activity by blocking the binding of LPS to toll-like receptor 4 and consequently inhibiting the phosphorylation of p38 mitogen-activated protein kinase and the nuclear translocation of NF-κB (52). Collectively, these findings suggest that dietary supplementation with CAD may enhance the immune capabilities of growing male minks.

The intestinal tract is the principal site for digestion, absorption, and immune response, serving as the barrier against invading pathogens (53). The experiment demonstrated that dietary supplementation with CAD at 100, 200, and 500 mg/kg increased the jejunum SIgA content, and CAD at 200 and 400 mg/kg resulted in a higher jejunum IL-10 content. Additionally, CAD at 100 mg/kg reduced the jejunum TNF-*α* content in growing males. Dai et al. (54) reported that dietary supplementation with AMP increased the intestinal SIgA content. Tang et al. (55) indicated that AMP could significantly downregulates the intestinal TNF-α level and increased the IL-10 level. SIgA is the predominant immunoglobulin in the intestinal mucosa, capable of neutralizing toxins, preventing the invasion of foreign pathogenic microorganisms, and regulating the intestinal microbiota (56). Inflammation in the body is regulated by a balance of pro-inflammatory and anti-inflammatory cytokines. Abnormal expressions of pro-inflammatory cytokines during disease can lead to pathological injury and weakened immune function (57). TNF-α is recognized as a pro-inflammatory cytokine involved in the development of ulcerative colitis (58). IL-10 is a potent inhibitor of immune and inflammatory responses (59). CAD can inhibit the expression of inflammatory factors, likely by binding to lipopolysaccharides (LPS), which disrupts the interaction between LPS and toll-like receptor 4 (TLR4). This interaction inhibition subsequently suppresses downstream TLR4-related signaling pathways, thereby enhancing immune function (60). Meanwhile, the significantly decreased intestinal gene expression level of nf-κb p65 in fish fed CAD diet indicated that CAD probably blocked the nf-κb-triggered overexpression of intestinal inflammatory cytokines (61). Thus, CAD in the diet can reduce intestinal inflammation-induced damage and strengthen intestinal immunity.

Maintaining the homeostasis of the intestinal microbiota in animals is crucial for nutrient absorption and intestinal health (62). The homeostasis is maintained through intricate interactions among the various components of the microbiota (63, 64). In our study, minks that received CAD at 100 mg/kg of diet showed a higher alpha diversity in their intestinal microbiota. This result is consistent with the findings of Tan et al. (65) in weaned piglets. The diversity and richness of the intestinal microbiota are closely linked to overall health, inflammation is known to decrease the diversity and richness of bacterial populations in the colon (66). AMPs demonstrate targeted antimicrobial activity and also have the ability to modulate intestinal pH value. By lowering the pH of the gut environment, they effectively hinder the colonization and invasion by harmful microorganisms and, concurrently, promote the proliferation of beneficial probiotics (32). Consequently, it can be concluded that appropriate supplementation of CAD in the diet can enhance intestinal health and promote the diversity of the intestinal microbiota.

The study revealed that CAD significantly modulated the composition of the intestinal microbiota in growing male minks. Minks in the CAD groups displayed a decreased relative abundance of *Escherichia-Shigella* and *Mycoplasma*, and those in the groups receiving CAD at 100, 200, and 400 mg/kg exhibited a lower relative abundance of *Peptostreptococcaceae*. Tang et al. (67) reported that AMPs reduced the presence of *E. coli* in the intestines of piglets. *E. coli* can induce several intestinal morphological alterations, including increased crypt depth and diminished villus height (68). Research has demonstrated that *E. coli* infection significantly elevates the pro-inflammatory factors while reducing anti-inflammatory factors (69). In weaned piglets, *E. coli* exposure led to decreased AST/GOT and ALT/GPT, and significantly suppressed serum AKP activity (70). The high abundance of *Peptostreptococcaceae* been linked to colorectal cancer (71) and ulcerative colitis (72). Studies have also demonstrated a negative correlation between *Peptostreptococcaceae* and feed efficiency (73). Therefore, CAD reduces the fractional abundance of harmful bacteria in the gut and can contribute to improved health of minks. In this experiment, 400 mg/kg CAD resulted in an increase in the relative abundance of *Staphylococcus* in the gut. Ouyang et al. (17) found that high doses of AMPs can increase the relative abundance of *Streptococcus* in the gut, which may be the reason for the occurrence of diarrhea in piglets. While the 400 mg/kg dose led to an increase in *Staphylococcus*, this observation was not consistent across all studies, as the 500 mg/kg CAD supplementation did not result in a similar increase in this study. This discrepancy suggests that the impact of CAD on gut microbiota may be dose-dependent and context-specific, highlighting the need for further research to fully understand the mechanisms and optimal dosing strategies for CAD supplementation. AMPs can inhibit essential processes such as DNA replication, transcription, and expression by interacting with the genetic material of harmful bacteria. Additionally, they disrupt bacterial protein synthesis, thereby preventing the proliferation of these detrimental microbes (9). AMPs can also disrupt the cell membrane of harmful bacteria by forming pores and increasing membrane permeability, ultimately destroying membrane integrity. This sequence of actions inhibits bacterial growth or leads to the death of the bacteria (74). Therefore, CAD could modulate intestinal health associated with the gut microbiota.

## 5 Conclusion

The results demonstrated that dietary supplementation with CAD could improve growth performance, nutrient utilization, immunocompetence, intestinal morphology and flora in growing male minks. Specifically, the group supplemented with 100 mg/kg CAD exhibited an optimal serum immune response and gut microbial abundance, while the group receiving 200 mg/kg CAD showed the best growth performance, intestinal digestibility, and intestinal immunity. Based on these findings, the recommended supplementation of CAD in diets for growing male mink is 100 to 200 mg/kg.

## Data Availability

The original contributions presented in the study are included in the article/supplementary material, further inquiries can be directed to the corresponding author.
